# Integrated DNA and RNA extraction using magnetic beads from viral pathogens causing acute respiratory infections

**DOI:** 10.1038/srep45199

**Published:** 2017-03-23

**Authors:** Hui He, Rongqun Li, Yi Chen, Ping Pan, Wenjuan Tong, Xueyan Dong, Yueming Chen, Daojun Yu

**Affiliations:** 1 The Affiliated First Hospital of Hangzhou, Zhejiang Chinese Medical University, Hangzhou, China; 2 Department of Pathology, Zhoushan Hospital, Zhoushan, Zhejiang Province, China; 3 College of Basic Medicine, Zhejiang Chinese Medical University, Hangzhou, China; 4 Department of Clinical Laboratory, Hangzhou First People’s Hospital, Hangzhou, China

## Abstract

Current extraction methods often extract DNA and RNA separately, and few methods are capable of co-extracting DNA and RNA from sputum. We established a nucleic acid co-extraction method from sputum based on magnetic beads and optimized the method by evaluating influencing factors, such as the guanidinium thiocyanate (GTC) and dithiothreitol (DTT) concentrations, magnetic bead amount, incubation temperature, lysis buffer pH and RNA carrier type. The feasibility of the simultaneous nucleic acid co-extraction method was evaluated by amplifying DNA and RNA viruses from a single clinical specimen with a multiplex RT-qPCR method. Both DNA and RNA were most efficiently extracted when the GTC and DTT concentrations were 2.0 M and 80 mM, respectively, 20 μl magnetic beads were added, the incubation temperature was 80 °C, the pH was 8 or 9, and RNA carrier A was used. Therefore, we established a simple method to extract nucleic acids from two important respiratory viruses compared with other commercial kits. This magnetic beads-based co-extraction method for sputum followed by a multiplex RT-qPCR can rapidly and precisely detect DNA and RNA viruses from a single clinical specimen and has many advantages, such as decreased time, low cost, and a lack of harmful chemicals.

Acute respiratory infections (ARIs) are a worldwide problem that can develop into more severe diseases, such as pneumonia, which cause high morbidity and mortality, and can also give rise to significant financial burdens on healthcare systems. An estimated 1.4 million deaths caused by pneumonia occurred in 2010, and pneumonia was responsible for 18.3% of total child deaths among children younger than 5 years of age worldwide; therefore, ensuring a correct diagnosis and providing timely treatment are crucial to accelerate the reduction of morbidity and mortality caused by pneumonia^1^,^2^. An abundance of experimental and epidemiological studies have identified viruses as the pathogens underlying most ARIs^3^,^4^. The World Health Organization estimates that approximately 34 million children under 5 years of age are infected with respiratory syncytial virus (RSV) each year, which represents 22% of all causes of acute lower respiratory infections (ALRIs), and 69,000–199,000 children die of RSV-associated ALRIs^5^,^6^. Although most pediatric adenovirus (ADV) respiratory infections are considered milder, the disease can be severe in some children and can even result in substantial morbidity^7^. Adenoviruses have been reported to account for 2–5% of overall respiratory illnesses and 4–10% of pneumonia cases^8^. However, these causative agents cannot be differentiated based on clinical symptoms alone. The current gold standard diagnostic tests for pathogens causing ARIs have a long turnaround time of at least 48 hours for bacterial pathogens (bacteria culture) and even longer time requirements for viral pathogens (viral culture). Moreover, due to the diagnostic uncertainty for clinicians, antibiotics are often prescribed even when a bacterial infection is not confirmed, which contributes to be the thorny issue of antibiotic resistance^9^.

Therefore, the development of better diagnostic tools for respiratory tract infections will have a tremendous impact on the treatment of these pathologies^10^. Researchers have not ceased to develop new diagnostic technologies, and nucleic acid amplification protocols to detect many viral pathogens have become mainstream in diagnostic laboratories. Among these protocols, the multiplex real-time quantitative polymerase chain reaction (qPCR) is the most frequently performed method due to its advantages of high sensitivity, specificity and throughput^11^. The prerequisite to implement these technologies is the extraction of high quality nucleic acids^12^. Viral nucleic acids are primarily divided into deoxyribonucleic acid (DNA) and ribonucleic acid (RNA)^13^, which share equally important statuses in ARIs^11^. For example, RSV (RNA virus) and ADV (DNA virus) are both common viruses that cause pneumonia in children. Therefore, the development of a method for the simultaneous extraction of DNA and RNA and a multiplex RT-qPCR method to detect these two different viruses are practical and significant goals.

Although current nucleic acid extraction methods often extract DNA and RNA separately, there are many commercially available kits for co-extract RNA and DNA. Some of these are very simple to use e.g. column-based. Some of them are marketed as RNA only or DNA only but actually co-extract very efficiently. However, commercially available co-extraction kits often have many steps for that the nucleic acids need to be transferred many times during the process^14^, and these kits are designed for DNA and RNA extraction from plasma or materials rich in DNA and RNA^12^,^14^,^15^,^16^,^17^. In contrast, few protocols are available for the extraction of integrated DNA and RNA from sputum samples, which contain large amounts of factors such as mucoproteins which wrap up nucleic acids make it hard to be extracted, saccharides, and lipids which make it hard to extract pure nucleic acids from the sputum, especially fragile RNA^18^,^19^. The optimal protocol should not only offer nucleic acids in a high quality and quantity but also have advantages such as low time consumption, reduced hands-on time, and a low price. Recently, the extraction of nucleic acids using magnetic beads has become a popular approach^12^,^20^,^21^ due to the high potential for automation. However, few reports have investigated viral DNA/RNA co-extraction from sputum samples using magnetic beads.

Because multiplex RT-qPCR can simultaneously detect viral DNA and RNA^22^, co-extraction of DNA and RNA from sputum samples will provide a significant advantage in terms of both simplifying the detection process and improving the veracity of the diagnosis. Therefore, the development of a simple and convenient method to co-extract DNA and RNA from sputum samples is an important goal.

In this study, we described a rapid and specific detection method for two genera of respiratory viruses (ADV and RSV) from the same sample. The method involves two steps: DNA and RNA co-extraction from the sputum and a multiplex RT-PCR. We optimized and evaluated the feasibility of a simultaneous nucleic acid co-extraction method based on magnetic beads and the amplification of DNA and RNA viruses from sputum and developed a multiplex RT-qPCR method to detect DNA and RNA viruses in a single clinical specimen.

## Results

### Guanidinium thiocyanate (GTC) concentration

**Figure 1:**
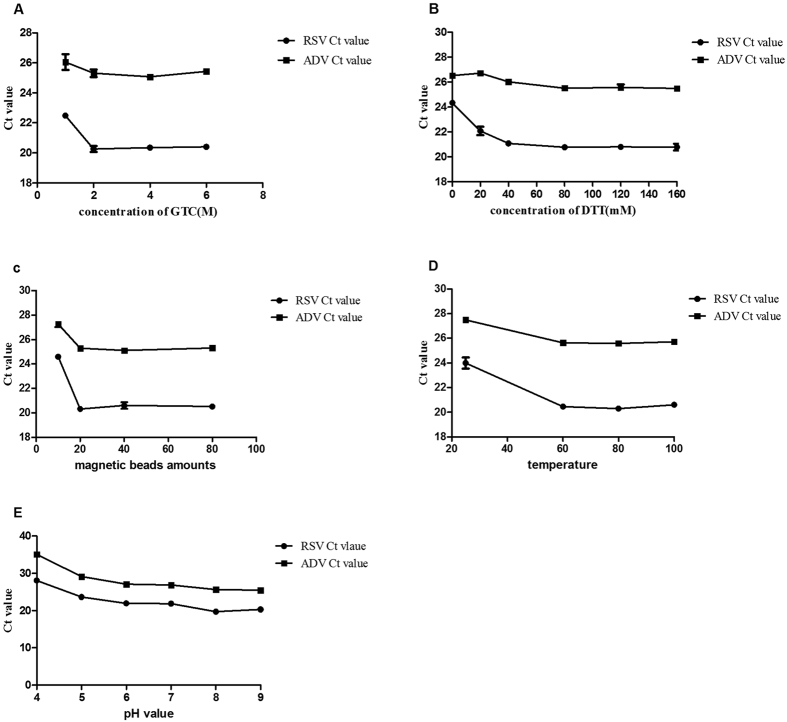
The Ct values of RNA (RSV) or DNA (ADV) obtained from the duplex RT-qPCR based on the assessment of different influencing factors. For GTC concentrations (**A**) for RSV, there were no statistical differences among groupA2, A3 and A4, but there were statistical differences among group A2, A3, A4 and group A1 (F = 51.29, *P* = 0.00); for ADV, the differences among all the groups weren’t statistically significant (F = 1.94, *P* = 0.20). For DTT concentrations (**B**) for RSV, there were no statistical differences among group B3, B4, B5 and B6, but there were statistical differences among group (B3, B4, B5, B6), group B2 and group B1 (F = 38.84, *P* = 0.00); for ADV, there were no statistical differences among group B3, B4, B5, B6, but there were statistical differences among group (B3, B4, B5, B6), group B2, group B1 (F = 10.18, *P* = 0.00). For magnetic bead amounts (**C**) for RSV, there were no statistical differences among group C2, C3 and C4, but there were statistical differences among group C2, C3, C4 and group C1 (F = 185.32, *P* = 0.00); for ADV, there were no statistical differences among group C2, C3 and C4, but there were statistical differences among group C2, C3, C4 and group C1 (F = 42.11, *P* = 0.00). For temperatures (**D**) for RSV, there were no statistical differences among group D2, D3 and D4, but there were statistical differences between group D2, D3, D4 and group D1 (F = 44.56, *P* = 0.00); for ADV, there were no statistical differences among group D2, D3 and D4, but there were statistical differences between group D2, D3, D4 and group D1 (F = 77.52, *P* = 0.00). For pH (**E**) for RSV, there was no statistical differences between group E5 and E6, and so was between group E3 and E4, but there were statistical differences among group (E5, E6), group (E3, E4), group E1 and group E2 (F = 85.36, *P* = 0.00); for ADV, there was no statistical differences between group E5 and E6, and so was between group E3 and E4, but there were statistical differences among group (E5, E6), group (E3, E4), group E1 and group E2 (F = 1370.60, *P* = 0.00).

The GTC concentration in the lysis buffer ranged from 1.0 M to 6.0 M while the other basic extraction buffer components were maintained as the method described in ‘selection of basic extraction buffer components’ part. The Ct values of RSV and ADV were shown in Fig. 1 and Table S1. For Ct values of RSV (RNA), group A1 > group A4 > group A3 > group A2, there were statistical differences among group A2, A3, A4 and group A1 through SNK test (F = 51.29, *P* = 0.00), and so was significant difference between group A1 and group A2 through t-test (*P* < 0.05). The optimal RNA extraction efficiency for RSV (RNA) was obtained when the GTC concentration was 2.0 M (group A2). Although the minimum Ct value for ADV (DNA) occurred at a GTC concentration of 4.0 M, the differences among all the groups weren’t statistically significant through SNK test (F = 1.94, *P* = 0.20). Therefore, a GTC concentration of 2.0 M was proposed to better extract both RSV (RNA) and ADV (DNA) (Table 1).

### Dithiothreitol (DTT) concentration

The DTT concentration in the lysis buffer ranged from 0 mM to 160 mM while the other basic extraction buffer components were maintained as the method described in ‘selection of basic extraction buffer components’ part. The Ct values of RSV and ADV were shown in Fig. 1 and Table S2. For Ct values of RSV (RNA), group B1 > group B2 > group B3 > group B5 > group B6 > group B4, there were statistical differences among group (B3, B4, B5, B6), group B2 and group B1 through SNK test (F = 38.84, *P* = 0.00), so was significant difference between group B2 and group B4 through t-test (*P* < 0.05). The optimal RSV (RNA) extraction efficiency was obtained at a DTT concentration of 80 mM (group B4), whereas the optimal DTT concentration for ADV (DNA) was 160 mM. For Ct values of ADV (DNA), group B2 > group B1 > group B3 > group B5 > group B4 > group B6, there were statistical differences among group (B3, B4, B5, B6), group B2, group B1through SNK test (F = 10.18, *P* = 0.00). Since little difference in the Ct values for ADV was observed between group B4 (80 mM) and group B6 (160 mM) through t-test (*P* > 0.05), the 80 mM DTT concentration exhibited high extraction efficiencies for both RSV (RNA) and ADV (DNA) (Table 1).

### Magnetic bead amount

The amount of magnetic beads used in this method ranged from 10 μl to 80 μl while the other basic extraction buffer components were maintained as the method described in ‘selection of basic extraction buffer components’ part. The Ct values of RSV and ADV were shown in Fig. 1 and Table S3. For Ct values of RSV (RNA), group C1 > group C3 > group C4 > group C2, there were statistical differences among group C2, C3, C4 and group C1 through SNK test (F = 185.32, *P* = 0.00, so was between group C2 and group C1 through t-test (*P* < 0.05). The minimum Ct value occurred in group C2 (20 μl). For Ct values of ADV (DNA), group C1 > group C4 > group C2 > group C3, there were statistical differences among group C2, C3, C4 and group C1 through SNK test (F = 42.11, *P* = 0.00). Although the extraction efficiency was optimal when 40 μl of magnetic beads was added, the difference between group C2 and group C3 was insignificant through t-test (*P* > 0.05). Therefore, an ideal simultaneous DNA and RNA extraction outcome was obtained with the addition of 20 μl of magnetic beads (Table 1).

### Effect of the incubation temperature and pH value

The incubation temperature ranged from room temperature to 100 °C while the other basic extraction buffer components were maintained as the method described ‘selection of basic extraction buffer components’ part. The Ct values of RSV and ADV are shown in Fig. 1 and Table S4. For Ct values of RSV (RNA), group D1 > group D4 > group D2 > group D3, there were statistical differences among group D2, D3, D4 and group D1 through SNK test (F = 44.56, *P* = 0.00), so was between group D3 and group D1 through t-test (*P* < 0.05). For Ct values of ADV (DNA), group D1 > group D4 > group D2 > group D3, there were statistical differences among group D2, D3, D4 and group D1 through SNK test (F = 77.52, *P* = 0.00), so was between group D3 and group D1 through t-test (*P* < 0.05). The minimum Ct value occurred in group D3 (80 °C) for both RSV (RNA) and ADV (DNA). Based on these results, we decided to use an incubation temperature of 80 °C to extract the nucleic acids (Table 1).

The pH of the lysis buffer was adjusted with 1 N hydrochloric acid and 1 N sodium hydroxide to values ranging from 4.0 to 9.0. The Ct values obtained for RSV and ADV were shown in Fig. 1 and Table S5. For RSV (RNA), the Ct values were as follows: group E1 > group E2 > group E3 > group E4 > group E6 > group E5, there were statistical differences among group (E5, E6), group (E3, E4), group E1 and group E2 through SNK test (F = 85.36, *P* = 0.00). The difference between group E5 and group E6 was insignificant through t-test (*P* > 0.05). The RSV (RNA) extraction efficiency was optimal at a pH of 8. For ADV (DNA), the Ct values were as follows: group E1 > group E2 > group E3 > group E4 > group E6 > group E5, there were statistical differences among group (E5, E6), group (E3, E4), group E1 and group E2 through SNK test (F = 1370.60, *P* = 0.00). The difference between group E5 and group E6 was insignificant through t-test (*P* > 0.05). Therefore, we propose that a pH of 8 or 9 is optimal for the extraction of both RSV (RNA) and ADV (DNA) (Table 1).

### Comparison of nucleic acid extraction methods

**Figure 2:**
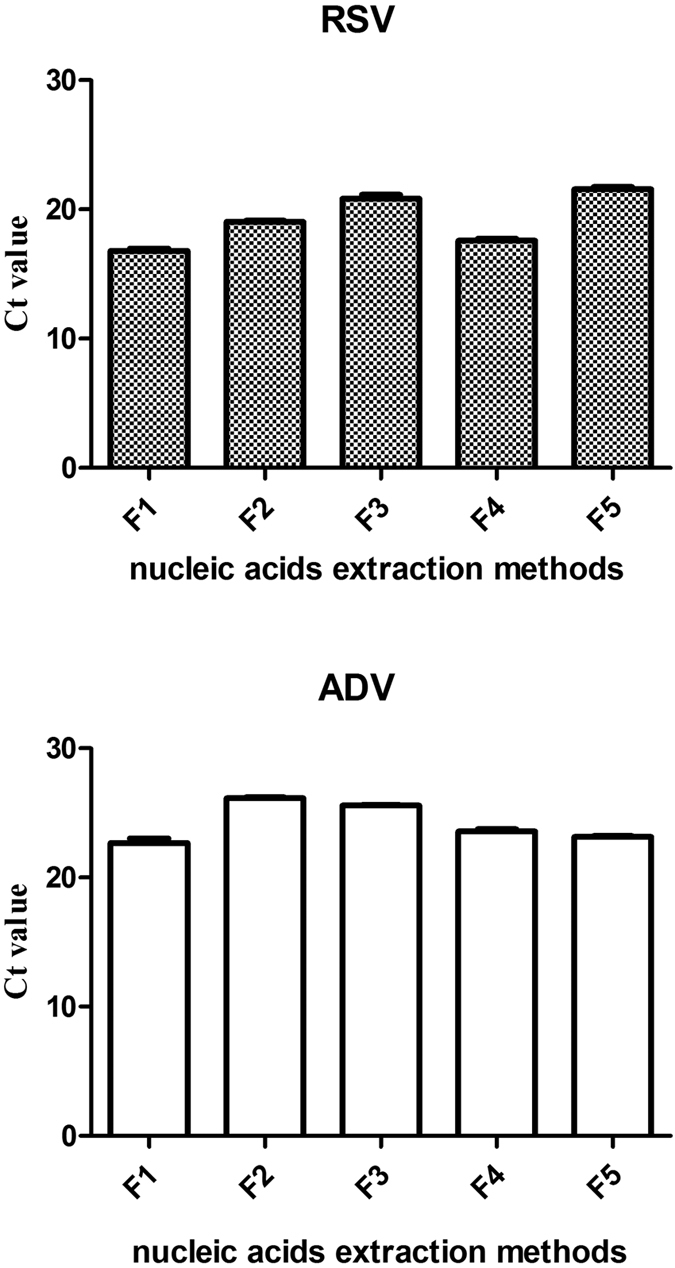
The comparison of Ct values obtained from the different nucleic acid extraction methods. F1: RNA carrier A; F2:RNA carrier B; F3: no RNA carrier; F4: TIANGEN; F5: TaKaRa RNA/DNA extraction kits that extract RNA and DNA separately. For different viral nucleic acid extraction methods, For Ct values of RSV (RNA), there were statistical differences among all the groups through SNK test (F = 106.73, *P* = 0.00). For Ct values of ADV (DNA), the differences among groups F5, F4 and F1 were not statistically significant, but there were statistical differences among group F2, F3 and group (F1, F4, F5) (F = 75.14, *P* = 0.00).

The extraction efficiencies of magnetic beads with or without RNA carriers and commercial kits were compared using Ct values obtained from a duplex RT-qPCR. The Ct values of ADV and RSV were shown in Fig. 2 and Table S6. For RSV (RNA), the Ct values were as follows: group F5 > group F3 > group F2 > group F4 > group F1, there were statistical differences among all the groups (F = 106.73, *P* = 0.00). The extraction efficiency was highest using the magnetic bead method with RNA carrier A, followed by the TIANamp Virus DNA/RNA Kit, the magnetic bead method with RNA carrier B, the magnetic bead method without an RNA carrier, and the TaKaRa RNA extraction kit. For ADV (DNA), the Ct values were as follows: group F3 > group F2 > group F4 > group F5 > group F1, the differences among groups F5, F4 and F1 were not statistically significant, but there were statistical differences among group F2, F3 and group (F1, F4, F5) (F = 75.14, *P* = 0.00). The highest extraction efficiency was obtained using the magnetic bead method with RNA carrier A (Table 1), followed by the TaKaRa RNA extraction kit, TIANamp Virus DNA/RNA Kit, magnetic bead method with RNA carrier B, and magnetic bead method without an RNA carrier (group F3). These results suggested that the magnetic bead method with the acryl carrier had the same efficiency as the TIANamp Virus DNA/RNA Kit for RNA and DNA co-extraction and the same efficiency as the TaKaRa DNA extraction kit for DNA extraction. The nucleic acid extraction efficiency of the magnetic bead method without an RNA carrier was lower than the efficiency of the commercial kits.

## Discussion

Great efforts have been made to develop fast, high-throughput, and cost-effective viral nucleic acid co-extraction methods. The main chemical constituents for the co-extraction of RNA and DNA are GTC, DTT, sodium dodecyl sulfate (SDS), and β-mercaptoethanol^12^,^13^,^15^. In the 1990s, I. Casas, L established the “GuSCN-DNA/RNA” extraction method to extract viral RNA and DNA from cerebrospinal fluid, this method was the basis for most subsequent co-extraction methods^15^. We extracted either DNA or RNA from sputum more efficiently by combining these chemical constituents with magnetic beads. In our previous study, we also used SDS and β-mercaptoethanol to compound the viral lysis buffer. However, SDS can easily form crystals, which introduces inconvenience during sample processing, and β-mercaptoethanol has a disagreeable odor and is a risk for human health and an environmental pollutant. Therefore, we determined that GTC, DTT, glycogen, and sodium citrate were sufficient and appropriate components of a lysis buffer to simultaneously extract DNA and RNA from sputum.

GTC is a strong denaturant that can inhibit RNase and DNase activities and separate nucleic acids and nucleoprotein, which preserves RNA and DNA integrity^12^,^15^. Several studies have shown that neither DNA nor RNA adsorbs onto the surfaces of magnetic beads if the lysis buffer lacks GTC^12^. This study showed that the highest extraction efficiency occurred with a 2.0 M GTC concentration based on the DNA and RNA co-extraction yields. The different GTC concentrations had little effect on the extraction efficiency. Notably, GTC crystallized when its concentration was 6.0 M, which introduced inconvenience during sample processing. DTT not only causes proteins to lose additional activity but also has a well-known ability to liquefy sputum^19^,^23^. Therefore, the sputum did not require prepossessing when DTT was included in the nucleic acid extraction process. Glycogen was used to facilitate nucleic acid precipitation^15^. Automatization of traditional nucleic acid extraction methods is difficult to implement due to the centrifugal steps^24^. The development of magnetic bionanoparticles has greatly promoted the nucleic acid extraction automatization process^12^,^25^. A large number of studies have shown that magnetic beads have the ability to absorb DNA and RNA^12^,^26^,^27^, and their adsorption capacity can be greatly enhanced with the appropriate concentrations of some solvents, including GTC. The reasons for this enhancement may be as follows^28^,^29^: anions are present on the surfaces of the magnetic beads and nucleic acids, and GTC or ammonium can function as a bridge between the nucleic acids and magnetic beads^30^; the high concentration of saline ions dehydrate the water layer on the surfaces of both nucleic acids and magnetic beads by forming hydrated ions, which prompt the magnetic beads to adsorb nucleic acids; and GTC makes the double-stranded DNA become single-stranded because the basic group of the single-stranded DNA and the hydroxyl group on the magnetic bead surface form a chemical bond that makes the adhesion stronger. Therefore, DNA and RNA are barely adsorbed onto the magnetic bead surface without GTC.

Although magnetic beads play an irreplaceable role in this extraction method, the extraction efficiency does not necessarily increase with the increase in the magnetic beads. The magnetic beads used in this study were modified with large quantities of hydroxyl. In a high salt environment, magnetic beads can absorb nucleic acids by separating them from proteins and other impurities. The results revealed that the extraction efficiency was reduced by the addition of an overabundance of magnetic beads; the nucleic acids on the surfaces of the magnetic beads may have been lost during the washing step because the magnetic beads overbound the extraction reagent, resulting in a decrease in the eluted nucleic acid in the final step. In this study, 20 μl magnetic beads were sufficient to guarantee a high extraction efficiency.

Many nucleic acid extraction methods are performed at room temperature^12^,^31^, whereas other studies have shown that more DNA and RNA were released at increased temperatures. Our experimental data showed that RNA degradation was not obvious at 60–100 °C, whereas incubation at room temperature resulted in a lower RNA extraction efficiency. The pH of the mixture plays an important role in nucleic acid extraction. As shown by our data, few differences were observed in the adsorption capacity of the magnetic beads under alkaline conditions, whereas the adsorption capacity decreased dramatically under acidic conditions, particularly when the pH was less than 5. These results might have occurred because proteins or amino acids adhered to the surfaces of the magnetic beads, which carry a positive charge, when the pH was too low, which affected nucleic acid binding to the magnetic beads.

This co-extraction method based on magnetic beads without carrier RNA met the requirements of a multiple RT-qPCR assay; however, the extraction efficiency was lower than the extraction efficiencies of the commercial kits. The addition of RNA carrier greatly improved the extraction efficiency and reached a higher level than the extraction efficiencies for the commercial kits for both RNA and DNA, possibly because the RNA carrier had a good effect on nucleic acid precipitation.

## Materials and Methods

### Ethics Statement

The use of the retrospective clinical specimens was approved by the Medical Ethics Committee of Hangzhou First People’s Hospital (reference number 201526). Informed consent was obtained from the parents of each patient for the collection and publication of de-identified information. We strictly followed the relevant regulations and institutional polices.

### Reagents and enzymes

All chemicals used in this study were analytical grade and did not require further purification. All solutions were prepared with DEPC (diethyl pyrocarbonate)-treated water. The magnetic beads (10 mg/ml) were purchased from Beaver Nano-Technologies Co., Ltd. GTC was purchased from Amersco. Ethanol was supplied by Hangzhou Longshan Chemical Co., Ltd. The acryl carrier was purchased from Beijing BLKWB Biotechnology Co., Ltd. DTT was purchased from Merck. Glycogen was obtained from Axygen. The One Step PrimeScript RT-PCR kit for Perfect Real-Time was purchased from TaKaRa. The MiniBEST Universal RNA Extraction kit and the TaKaRa MiniBEST Universal Genomic DNA Extraction kit Ver5.0 were purchased from TaKaRa. The TIANamp Virus DNA/RNA Kit was purchased from Tiangen Biotech (Beijing) Co., Ltd.

### Biological specimens

Thirty clinical sputum specimens in which RSV or ADV was detected by a direct immunofluorescence assay were obtained from hospitalized children with suspected acute respiratory infections in Hangzhou First People’s Hospital. All specimens were mixed and homogenized with 0.1% DTT and then diluted to 50 ml with 0.9% normal saline (NS) and a number of 200 ul aliquots taken from the 50 mls (each aliquot in a 1.5 ml centrifuge tube containing 200 μl of diluted sputum). Three of the aliquots were screened to confirm the presence of RSV and ADV by qPCR/RT-qPCR and sequencing. All samples were stored in a −80 °C freezer prior to the optimization steps.

### Nucleic acid extraction

#### Selection of basic extraction buffer components

DNA and RNA were simultaneously extracted from mixed sputum specimens using magnetic beads. Briefly, a 200 μl sample was centrifuged at 12,000 × g at 4 °C for 5 minutes; then, the supernatant was discarded, and 300 μl of lysis buffer^15^ (2 M guanidinium thiocyanate, 80 mM dithiothreitol, 25 mM sodium citrate, and 20 μg/ml of glycogen, pH at 6) was immediately added to the tube and vigorously vortexed for 15 seconds. The lysates were subsequently incubated for 10 min at 80 °C. After cooling to room temperature, an equivalent volume of 100% ethanol and 20 μl of magnetic beads (10 mg/ml) were added to the tube and mixed well then, the mixture was incubated for 5 min at room temperature. The supernatant was discarded, and the magnetic beads which have absorbed nucleic acids were collected using a DynaMag^TM^-2 Magnet (Life Technologies, USA). The magnetic beads were washed twice with 70% ethanol. After adding 50 μl of RNase-free water which represents elution of the nucleic acids off the beads, the magnetic bead mixture was incubated for 5 min. Finally, the supernatant was transferred to a new tube and stored at −30 °C. RSV or ADV was detected by duplex RT-qPCR.

### Optimization of the influencing factors

To optimize this extraction method, the GTC and DTT concentrations, magnetic bead amount, incubation temperature, and lysis buffer pH were evaluated as influencing factors. Each factor (e.g., GTC) was optimized while the other factors were maintained as the method described in’Selection of basic extraction buffer components’part. The tested GTC concentrations ranged from 1.0 M to 6.0 M as follows: group A1 (1.0 M), group A2 (2.0 M), group A3 (4.0 M), and group A4 (6.0 M). The tested DTT concentrations ranged from 0 mM to 160 mM as follows: group B1 (0 mM), group B2 (20 mM), group B3 (40 mM), group B4 (80 mM), group B5 (120 mM), and group B6 (160 mM). The tested magnetic bead amounts ranged from 10 μl to 80 μl as follows: group C1 (10 μl), group C2 (20 μl), group C3 (40 μl), and group C4 (80 μl). The tested incubation temperatures ranged from room temperature to 100 °C as follows: group D1 (room temperature), group D2 (60 °C), group D3 (80 °C), and group D4 (100 °C). The tested pH values ranged from 4 to 9 as follows: group E1 (pH 4), group E2 (pH 5), group E3 (pH 6), group E4 (pH 7), group E5 (pH 8), and group E6 (pH 9). Each group was performed in parallel and triplicate with the other steps as described in ‘Selection of basic extraction buffer components’ part.

### Comparison with RNA/DNA co-extraction commercial kits

After the extraction method was optimized, we also evaluated the effect of two RNA carriers on the nucleic acid extraction yield using this method as follows: group F1 (RNA carrier A) and F2 (RNA carrier B). The results were compared with a viral RNA/DNA co-extraction commercial kit (TIANGEN) (group F4) and TaKaRa RNA/DNA extraction kits that extracted RNA and DNA separately (group F5). For group F1, RNA carrier A was the acryl carrier (Beijing BLKWB Biotechnology Co., Ltd), the addition of RNA carrier A was 6 μl for per extraction. For group F2, RNA carrier B was obtained from the TIANamp Virus DNA/RNA Kit (Tiangen Biotech (Beijing) Co., Ltd), the addition of RNA carrier B was 6 μl for per extraction, and for group F3 (no RNA carrier), the nucleic acids were extracted using the magnetic beads-based method without an RNA carrier. A 200 μl sample was used in each group, and the extracts were all eluted in 50 μl RNase-free water. The nucleic acid extraction yields were analyzed using duplex RT-qPCR.

### Duplex RT-qPCR

A duplex RT-qPCR was designed to determine whether the viruses (RSV and ADV) were present. The assays were performed in a 96-well optical reaction plate (Applied Biosystems 7500, Foster City, CA, USA) in a total volume of 25 μl containing 12.5 μl of 1× One Step RT-PCR buffer III, 0.5 μl of TaKaRa Ex TaqHS (5 U/μl), 0.5 μl of PrimeScript RT Enzyme Mix II, 0.5 μl of the sense primer (10 μmol/L), 0.5 μl of the antisense primer (10 μmol/L) and 0.5 μl of the RSV and ADV probes. All experiments were performed in accordance with we previously developed^32^.

The RSV and ADV primers and probes were designed based on available nucleotide sequence data from the GenBank database of the National Center for Biotechnology Information (NCBI) using the primer design tool. A nucleotide BLAST search (NCBI) analysis was used to verify the specificity of the primer and probe sequences. The primers and probes were shown in Table 2. The primers used in the multiplex RT-qPCR were synthesized by Life Technologies (Shanghai, China), and AllGlo probes were obtained from Huirui Biocompany (Shanghai, China)^33^. The experimental PCR protocol was as follows: reverse transcription step at 42 °C for 5 min, an initial denaturation step at 95 °C for 10 s, followed by 40 cycles of 95 °C for 10 s and an annealing extension step at 60 °C for 40 s. The Ct (threshold cycle) values in this experiment were calculated automatically by the SDS 2.4 software (Applied Biosystems). A low Ct value indicated a high amount of RNA or DNA.

### Data analysis

The factors influencing the magnetic bead method, including the GTC and DTT concentrations, magnetic bead amount, incubation temperature, and lysis buffer pH were analyzed though single-factor experiments. The analysis was carried out on Ct values. The comparison of differences caused by these influencing factors in the magnetic bead-based method and differences among the different extraction methods (magnetic beads, TIANGEN, and TaKaRa) were determined by the Student-Newman-Keuls (SNK) test of analysis of variance in SPSS Statistics 17.0 (SPSS Inc. Chicago, IL, USA). The comparisons between two groups were analyzed by using t-test. In all cases, the confidence interval was set at 95%.

## Conclusion

This magnetic bead-based method optimized factors that influenced the extraction method and the added RNA carrier and thus offered improved DNA and RNA Co-extraction efficiency from sputum samples. The time required was reduced, and the method facilitated sample preparation for the PCR assay; the whole sample preparation and nucleic acid isolation process could be finished in 30 min. The use of magnetic beads and the avoidance of centrifugal steps provide the foundation for the automation of RNA and DNA co-extraction. The simple procedure and the avoidance of organic solvents with disagreeable odors make this method attractive for use in routine diagnostic laboratories, especially for respiratory pathogen detection by multiplex RT-qPCR. While this is a preliminary study, the further validation on a panel of clinical samples should be done in a future study.

## Additional Information

**How to cite this article:** He, H. *et al*. Integrated DNA and RNA extraction using magnetic beads from viral pathogens causing acute respiratory infections. *Sci. Rep.* **7**, 45199; doi: 10.1038/srep45199 (2017).

**Publisher's note:** Springer Nature remains neutral with regard to jurisdictional claims in published maps and institutional affiliations.

## Supplementary Material

Supplementary Tables

## Figures and Tables

**Table 1:** Optimal quantities and equivalent Ct values for both virus of viral nucleic acid extraction.

Parameters/Assays	Value	RSV Ct	ADV Ct
GTC concentrations (M)	2	20.35 ± 0.30	25.07 ± 0.20
DTT concentrations (mM)	80	20.78 ± 0.14	25.52 ± 0.16
magnetic beads (μl)	20	20.31 ± 0.20	25.29 ± 0.15
Incubation temperature (°C)	80	20.30 ± 0.28	25.59 ± 0.12
pH	8 or 9	19.71 ± 0.55 or 20.33 ± 0.72	25.66 ± 0.14 or 25.47 ± 0.30
RNA carriers A (μl)	6 μl	16.80 ± 0.30	22.67 ± 0.57

It is average Ct values that are presented.

**Table 2:** Sequences of the primers and probes used in this study.

	Sequences (5′-3′)	Amplified fragment length	
RSV	Primer F	GCACCGCCAAGACACTAGAA	179
(RNA	Primer R	GTGGTTTGCCGAGGCTATGA	
Virus)	Probe	MAR -GGA CCT GGG ACA CTC TCA ATC ATC T- MAR	
ADV	Primer F	TAAGACTCTCGTCCATTTGGTCA	154
(DNA	Primer R	CTTAACATCGCCGCCAAGGAG	
Virus)	Probe	SAT –CACAATCTTCTTGTTGTCCAGCTTGG- SAT	
