# Supplementary material for: Integrated DNA and RNA extraction using magnetic beads from viral pathogens causing acute respiratory infections
Source: Sci Rep. 2017 Mar 23;7:45199. doi: 10.1038/srep45199 (PMC5362898; doi:10.1038/srep45199)
Supplement: Supplementary Tables [file srep45199-s1.pdf]

Supplementary Tables to:

Integrated DNA and RNA extraction using magnetic beads from viral pathogens causing acute respiratory infections

*Hui He[1,4], *Rongqun Li[3], Yi Chen[1,2], Ping Pan[1,2], Wenjuan Tong[2], Xueyan Dong[2], Yueming Chen[2], Daojun Yu[1,2]

[1] The Affiliated First Hospital of Hangzhou, Zhejiang Chinese Medical University, Hangzhou, China;

[2] Department of Clinical Laboratory, Hangzhou First People's Hospital, Hangzhou, China;

[3] College of Basic Medicine, Zhejiang Chinese Medical University, Hangzhou, China.

[4] Department of Pathology, Zhoushan Hospital, Zhoushan, Zhejiang Province, China.

*Equal contributors.

Supplementary Tables

Table 1. Sequences of the primers and probes used in this study

	Sequences (5'-3')		Amplified fragment length
RSV	Primer F	GCACCGCCAAGACACTAGAA	179
(RNA	Primer R	GTGGTTTGCCGAGGCTATGA	
Virus)	Probe	MAR -GGA CCT GGG ACA CTC TCA ATC ATC T- MAR	
ADV	Primer F	TAAGACTCTCGTCCATTTGGTCA	154
(DNA	Primer R	CTTAACATCGCCGCCAAGGAG	
Virus)	Probe	SAT –CACAATCTTCTTGTTGTCCAGCTTGG- SAT	

Table 2 qPCR-based Ct value evaluation of viral nucleic acid extraction based on different GTC concentrations

	RSV Ct	ADV Ct
A1	22.48±0.10	26.06±0.91
A2	20.26±0.34	25.31±0.43
A3	20.35±0.30	25.07±0.20
A4	20.41±0.22	25.43±0.21
F	51.29	1.94
P	0.00	0.20

The GTC concentrations ranged from 1.0 M to 6.0 M as follows: group A1 (1.0 M), group A2 (2.0 M), group A3 (4.0 M), and group A4 (6.0 M).

Table 3 qPCR-based Ct value evaluation of viral nucleic acid extraction based on different DTT concentrations

	RSV Ct	ADV Ct
B1	24.35±0.39	26.53±0.32
B2	22.08±0.58	26.72±0.29
B3	21.08±0.29	26.03±0.09
B4	20.78±0.14	25.52±0.16
B5	20.81±0.35	25.58±0.41
B6	20.78±0.46	25.50±0.36
F	38.84	10.18
P	0.00	0.00

The DTT concentrations ranged from 0 mM to 160 mM as follows: group B1 (0 mM), group B2 (20 mM), group B3 (40 mM), group B4 (80 mM), group B5 (120 mM), and group B6 (160 mM).

Table 4 qPCR-based Ct value evaluation of viral nucleic acid extraction based on different magnetic bead amounts

	RSV Ct	ADV Ct
C1	24.59±0.02	27.25±0.41
C2	20.31±0.20	25.29±0.15
C3	20.60±0.48	25.11±0.20
C4	20.53±0.09	25.32±0.25
F	185.32	42.11
P	0.00	0.00

The amount of magnetic beads ranged from 10 μl to 80 μl as follows: group C1 (10 μl), group C2 (20 μl), group C3 (40 μl), and group C4 (80 μl).

Table 5 qPCR-based Ct value evaluation of viral nucleic acid extraction at different temperatures

	RSV Ct	ADV Ct
D1	24.00±0.79	27.49±0.28
D2	20.46±0.32	25.64±0.10
D3	20.30±0.28	25.59±0.12
D4	20.62±0.21	25.73±0.17
F	44.56	77.52
P	0.00	0.00

The incubation temperature ranged from room temperature to 100 ℃ as follows: group D1 (room temperature), group D2 (60 ℃), group D3 (80 ℃), and group D4 (100 ℃).

Table 6 qPCR-based Ct value evaluation of viral nucleic acid extraction at different pH values

	RSV Ct	ADV Ct
E1	28.07±0.33	35.08±0.06
E2	23.66±0.68	29.17±0.15
E3	21.96±0.20	27.10±0.13
E4	21.89±±0.70	26.90±0.15
E5	19.71±0.55	25.66±0.14
E6	20.33±0.72	25.47±0.30
F	85.36	1370.60
P	0.00	0.00

The pH values ranged from 4 to 9 as follows: group E1 (pH 4), group E2 (pH 5), group E3 (pH 6), group E4 (pH 7), group E5 (pH 8), and group E6 (pH 9).

Table 7 qPCR-based Ct value evaluation of different viral nucleic acid extraction methods

	RSV Ct	ADV Ct
F1	16.80±0.30	22.67±0.57
F2	19.04±0.17	25.55±0.10
F3	20.83±0.55	26.15±0.12
F4	17.59±0.25	23.55±0.33
F5	21.55±0.31	23.13±0.13
F	106.73	75.14
P	0.00	0.00

F1: RNA carrier A; F2: RNA carrier B; F3: no RNA carrier; F4: TIANGEN; and F5: TaKaRa RNA/DNA extraction kits that extract RNA and DNA separately.
